# Construction of Shale Gas Oil-Based Drilling Cuttings Degrading Bacterial Consortium and Their Degradation Characteristics

**DOI:** 10.3390/microorganisms12020318

**Published:** 2024-02-02

**Authors:** Li Fan, Xianhe Gong, Quanwei Lv, Denghui Bin, Li’Ao Wang

**Affiliations:** 1 College of Resource and Safety Engineering, Chongqing University, Chongqing 400044, China; 2 Chongqing Academy of Ecology and Environmental Sciences, Chongqing 401336, China; gongxianhe0820@163.com (X.G.); bindenghui@foxmail.com (D.B.); 3 The Southwest Branch of the Chinese Academy of Environmental Sciences, Chongqing 401336, China

**Keywords:** shale gas, oil-based drilling cuttings, petroleum hydrocarbons, bacterial consortium

## Abstract

Oil-based drilling cuttings (OBDCs) contain petroleum hydrocarbons with complex compositions and high concentrations, which have highly carcinogenic, teratogenic, and mutagenic properties. In this study, three highly efficient petroleum hydrocarbon-degrading bacteria were screened from OBDCs of different shale gas wells in Chongqing, China, and identified as *Rhodococcus* sp. and *Dietzia* sp. Because of their ability to degrade hydrocarbons of various chain lengths, a new method was proposed for degrading petroleum hydrocarbons in shale gas OBDCs by combining different bacterial species. Results showed that the bacterial consortium, consisting of the three strains, exhibited the highest degradation rate for petroleum hydrocarbons, capable of degrading 74.38% of long-chain alkanes and 93.57% of short-chain alkanes, respectively. Moreover, the petroleum hydrocarbon degradation performance of the bacterial consortium in actual OBDCs could reach 90.60% in the optimal conditions, and the degradation kinetic process followed a first-order kinetic model. This study provides a certain technical reserve for the bioremediation of shale gas OBDCs.

## 1. Introduction

According to the US Energy Information Administration (EIA, 2016), global natural gas production will increase from 342 billion cubic feet per day (Bcf/d) in 2015 to 554 billion Bcf/d in 2040. Among them, the natural gas derived from shale resources increased from 42 Bcf/d in 2015 to 168 Bcf/d in 2040, constituting approximately 30% of the world’s total natural gas production [1]. However, the exploration and exploitation of shale gas fields result in the substantial production of oil-based drilling cuttings (OBDCs). According to China, it generates over 4 × 10^5^ m^3^ of OBDCs annually [2], with the Fuling shale gas region in Chongqing alone producing as much as 4 million tons annually [3]. Generally, they are characterized by petroleum hydrocarbons, heavy metals, and alkaline salts, leading to their classification as hazardous waste [4,5]. Therefore, effective disposal has emerged as a shared concern among countries involved in shale gas development.

Various methods, including landfilling [6], reinjection [7], stabilization/solidification [8], solvent extraction [9], and chemical cleaning [10], have been exploited for the treatment of OBDCs. However, these methods have not gained widespread adoption, primarily due to their high cost, low recovery rates, and high requirements for equipment performance. Additionally, treated OBDCs may still pose a risk of secondary pollution due to potential pollutant leakage [11]. Biodegradation technology emerges as a promising method for OBDC treatment, offering advantages such as low operating costs, minimal energy consumption, and reduced environmental risk [12]. This method involves the microbial metabolic process, decomposing petroleum hydrocarbon pollutants into carbon dioxide, water, and other harmless compounds [13].

Currently, many bacterial strains have been screened from contaminated soils and aqueous environments for petroleum hydrocarbon degradation, such as *Bacillus* sp. [14], *Pseudomonas* sp. [15], *Rhodococcus* sp. [16], and *Microbacterium* sp. [17]. Studies have explored the impact of factors such as temperature [18], C/N/P ratio [19], biosurfactant [20], and salinity [21] on the degradation efficiency of petroleum hydrocarbons by these bacterial strains. However, the petroleum hydrocarbons were difficult to degrade effectively over a single bacterial strain because of their complex composition [22]. Consequently, synergistic degradation of petroleum hydrocarbons by various strains has been proposed [23,24]. Some studies also reported the screening, identification, and application of petroleum hydrocarbons that degrade microorganisms from OBDCs in conventional oil/gas fields [10,25]. However, shale gas represents an unconventional energy source, with reservoir properties and exploitation methods differing significantly from conventional oil/gas fields [26]. Simultaneously, the evolving trend toward personalized drilling fluids may introduce additional variations in cutting composition [27]. Therefore, the biodegradation technology of conventional oil/gas fields cannot be directly applied to treating shale gas OBDCs. Currently, research on the biodegradation of shale gas OBDCs mainly focuses on single-well sites and lacks extensive field application examples. Furthermore, limited information is available regarding bacteria and bacterial consortia capable of efficiently and broadly degrading various shale gas OBDCs. Therefore, screening and constructing a bacterial consortium that can efficiently degrade petroleum hydrocarbons in multi-well sites has become a key scientific issue in using biodegradation technology to treat shale gas OBDCs.

This study aims to screen various bacteria capable of degrading petroleum hydrocarbons from shale gas (OBDCs) obtained from different drilling wells and establish a dominant bacterial consortium for the bioremediation of shale gas OBDCs. Therefore, the specific goals of this study are as follows: (1) conduct physical and chemical characterization of OBDCs samples; (2) screen and identify the highly efficient petroleum hydrocarbons degrading bacteria in OBDCs; (3) construct a highly efficient petroleum hydrocarbons degrading bacterial consortium; (4) optimize the degradation conditions for the degrading bacteria, including temperature, pH, salinity, N/P ratio, and diesel concentration; and (5) validate the degradation performance of the degrading bacterial consortium.

## 2. Materials and Methods

### 2.1. Sample and Diesel for Test

OBDCs samples were collected from shale gas drilling wells in Fuling (FL), Wulong (WL), Nanchuan (NC), and Dazu (DZ) of Chongqing. The collection involved using sterilized equipment, and the samples were carefully placed in individual sterilized opaque bags and bottles, each appropriately labeled. All samples were transported to the laboratory at 4 °C. Diesel was procured from a PetroChina gas station as the source of petroleum hydrocarbons in this study.

### 2.2. Physical and Chemical Characterization of Collected Samples

All samples underwent analysis for fundamental physical and chemical properties, encompassing pH (PHS-3G, Leici, Shanghai, China), water content, solid content (YH-C10002, Yingheng, Shanghai, China), and oil content (JLBG-126U, Jiguang, Jilin, China). The metal elements were detected by an X-ray fluorescence spectrometer (S8TIGER, Bruker, Karlsruhe, Germany). Total petroleum hydrocarbons (TPHs) were detected by gas chromatography (GC) (GC-2014, Shimadzu, Kyoto, Japan). The detection of BTEX and polycyclic aromatic hydrocarbons (PAHs) was carried out by gas chromatography-mass spectrometry (GC-MS) (TSQ 9000, Thermo Fischer Scientific, Waltham, MA, USA).

### 2.3. Culture Media

Mineral salt medium (MSM) contained the following components in 1 L of distilled water: trace element mixture (0.5 mL), KCl (0.7 g), KNO_3_ (2.0 g), MgSO_4_·7H_2_O (1.03 g), NaCl (20 g), KH_2_PO_4_ (1 g), and Na_2_HPO_4_ (1 g). The trace element mixture (pH 7.0) was composed of CaCl_2_ (26.49 mg/L), FeCl_3_·6H_2_O (50 mg/L), MnCl_2_·4H_2_O (5 mg/L), ZnSO_4_·7H_2_O (100 mg/L), and CuSO_4_ (7.83 mg/L). Luria-Bertani medium (LB) consisted of the following ingredients in 100 mL of distilled water: peptone (1 g), yeast extract (0.5 g), and NaCl (1 g). 15.0 g/L agar was added to the medium to prepare nutrient agar plates or slants. All media underwent autoclaving at 121 °C for 20 min.

### 2.4. Isolation and Screening of Strains

Inoculate 2 g of OBDCs sample into 100 mL of MSM medium and cultivate the mixture in a constant-temperature shaker at 28 °C and 180 rpm for 5 days. Subsequently, transfer 10% of the culture to fresh medium for the enrichment of oil-degrading bacteria, repeating this continuous process three times. Transfer 0.5 mL of the enriched liquid culture onto 1/2MSM+1/2LB solid agar plates for streaking to isolate predominant oil-degrading bacteria. The plates were incubated at 28 °C for 3–7 days, and then the colonies with distinctive morphology were selected and purified. Thus, potential oil-degrading bacteria were isolated from OBDCs.

The isolated strains were cultured in 1/2MSM+1/2LB medium, placed in a constant temperature shaker at 30 °C and 180 rpm for 1–5 d, and then centrifuged in a centrifuge at 5000 rpm for 5 min. Subsequently, they were centrifuged at 5000 rpm for 5 min, discarding the supernatant, and the bacteria were suspended in physiological saline. These steps were repeated three times to prepare the seed solution for the bacterial strains. The strain seed solution was added to MSM medium containing 1% diesel with OD_600_ = 1 and a 5% initial inoculum. The cells were cultured at 30 °C and 180 rpm for 7 days to determine their degradation efficiency, and the method was described in Section 2.9.

### 2.5. Identification of a Strain

#### 2.5.1. Morphological and Biochemical Characteristics

The morphological properties of these strains were examined by optical microscopy. We systematically analyzed these strains’ typical biochemical and physiological characteristics, following Bergey’s Manual of Determinative Bacteriology [28]. This analysis included assessments of Gram staining, gelatin liquefaction, starch hydrolysis, nitrate reduction, indole production, citrate utilization, and acid production from glucose.

#### 2.5.2. PCR Amplification of 16S rDNA

PCR amplification was performed for the 16S rDNA gene using universal primers 27F (5′AGAGTTTGATCCTGGCTCA3′) and 1492R (5′GGTTACCTTGTTACGACTT3′). The PCR amplification process involved an initial step at 94 °C for 5 min, followed by 30 cycles at 94 °C for 30 S, 55 °C for 30 S, and 72 °C for 2 min, with a final extension at 72 °C for 8 min, and then storage at 4 °C. Shanghai Sagon Biotech Ltd. (Shanghai, China) performed the sequencing of the PCR products. The BLAST tool of NCBI matched the DNA sequences with those in the database.

### 2.6. Construction of a Bacterial Consortium

The petroleum hydrocarbons that degrade bacteria were combined by non-repetitive permutations to construct the bacterial consortium. The OD_600_ of each strain in each combination was adjusted to 1, and the total inoculated amount of 2% was added to MSM medium containing 1% diesel. This mixture was cultured at 30 °C and 180 rpm for 7 d to determine the degradation efficiency of petroleum hydrocarbons. Refer to Section 2.9 for the determination method.

### 2.7. Optimization of Degradation Conditions for the Strains

Through single-factor experiments, we explored the optimal conditions for these strains to degrade petroleum hydrocarbons. This study investigated the effects of different temperatures (20 °C, 25 °C, 30 °C, 35 °C, 40 °C), salinities (0.1%, 0.5%, 1%, 2%, 3%), pH values (5.0, 6.0, 7.0, 8.0, 9.0), diesel concentrations (0.7%, 1.4%, 2.8%, 4.3%, 7%), and N/P ratios (0.3, 1, 2, 5, 10) on the degradation efficiency of the strain. After culturing in a shaker at 180 rpm for 7 d, the petroleum hydrocarbon degradation efficiency was measured, with 3 parallels set up for each group.

### 2.8. Validation of the Degradation Performance of the Bacterial Consortium

The screened, washed, dried, and sterilized river sand was added to the oil-based cuttings and stirred evenly so that the oil content in the final oil-based cuttings was 0.5%. Then, the single bacteria (NC-17) and bacterial consortium (NC-17/WL-3/FL-1) were added to the OBDCs at a 2% inoculation amount, and the MSM medium was added. The mixture was stirred every 2 d, and the bacterial solution was added every 20 d. During this period, the water content was maintained at 8~10%. Samples were taken every 10 d, and the degradation rate of petroleum hydrocarbons was determined by GC analysis.

### 2.9. GC Evaluation of Oil Biodegradation

Transferred the sample to the separator funnel and added 10 mL of n-hexane. Tightly sealed the bottle and shook vigorously for 3 min to allow for separation. Then, let the mixture stand for 3 min to allow for layering. Drained the water phase from the lower outlet of the separator funnel and transferred it to a clean beaker. Poured out the organic phase from the upper outlet of the separator funnel and saved it. Repeat the above operation to extract the sample again in the beaker. Subsequently, use 10 mL of n-hexane to wash out the residual solution in the separator funnel. Mix the two extracts and wash solutions in the same container, and then add Na_2_SO_4_ for dehydration. Filter the mixture through a 0.45 μm nylon filter.

The extracted samples underwent analysis using gas chromatography (GC, GC-2014, Shimadzu, Kyoto, Japan) equipped with a capillary column HP-5 (320 µm diameter, 30 m length, and 0.25 µm width). The injector and detector temperatures were set at 300 °C and 280 °C, respectively. Hydrogen served as the carrier gas (1.0 mL/min). The column temperature program was as follows: the initial temperature was maintained at 40 °C for 2 min, then raised to 300 °C at the heated rate of 15 °C/min, and maintained for 10 min. The biodegradation efficiency was calculated by Equation (1):

(1) $$BE=\left(1-A_{sample}/A_{control}\right)\times 100\%$$

where *BE* is the biodegradation efficiency, %; *A_sample_* and *A_control_* are the total peak area of the sample and control, respectively.

### 2.10. Statistical Analysis

All analyses were performed three times. The results were expressed as the mean and standard deviation of three repeated measurements. The Kolmogorov–Smirnov test was used to examine whether the data were normally distributed. The one-way analysis of variance (ANOVA) with Tuckey’s post hoc Test Calculator was utilized to analyze statistically significant differences using IBM SPSS Statistics (Version 29.0, IBM Inc., Armonk, NY, USA) statistical software. A significance level of *p* < 0.05 was applied to assess statistical significance.

## 3. Results and Discussion

### 3.1. Physical and Chemical Properties of Collected Samples

This study analyzed the physicochemical properties of different OBDCs, including parameters such as oil content, water content, solid content, and pH value (Table 1). BTEX, PAHs, TPH (Table 2), and metals (Table 3) were also tested. The preliminary analysis showed these samples had weak alkalinity. The water content was low, primarily ranging between 5.03% and 14.05%, which is favorable for subsequent treatment. The oil content varied from 6.60% to 11.16%, signifying a high potential for recycling. The solid content ranged from 73.6% to 85.57%, suggesting a substantial inorganic component in these samples (Table 1). In addition to the BTEX and PAHs in WL, PAHs in NC and Benzene in DZ were below the detection limits, with only a minimal quantity of BTEX and PAHs detected. Among these samples, the highest total petroleum hydrocarbons (TPHs) concentration was observed in WL, followed by NC, DZ, and FL (Table 2).

The metal analysis showed that FL had the highest total measured metal concentration, followed by NC, DZ, and WL (Table 3). In all samples, the concentrations of Ba and Sr were much higher than those of other metals, mainly attributed to the aggravates used in the drilling process. Previous studies have indicated that specific metal concentrations can positively impact microbial community structure and the diversity of carbon utilization [29]. Additionally, Li et al. demonstrated that heavy metal contamination significantly altered microbial community composition, α-diversity, and metabolic functions [30]. This study detected metals such as Mn, Cu, and Co in OBDCs. Prior studies have indicated that these metals can serve as electron acceptors in soils with limited oxygen content, potentially enhancing bioremediation activity. While oxygen typically functions as the primary electron acceptor under aerobic conditions, microorganisms can utilize Co and Mn as alternative electron acceptors in anaerobic environments [31]. Microorganisms can produce biosurfactants that reduce surface and interfacial tension. This process expands the contact area between microorganisms and hydrocarbons, thereby enhancing the efficiency of microbial degradation [32]. Makkar et al. showed that adding different metals to the medium could significantly affect bacterial growth and biosurfactant production [33]. Similarly, Kiran et al. found that biosurfactant production by marine actinobacteria increased with increasing metal concentrations [34].

### 3.2. Isolation, Screening, and Identification of Strains

**Figure 1:**
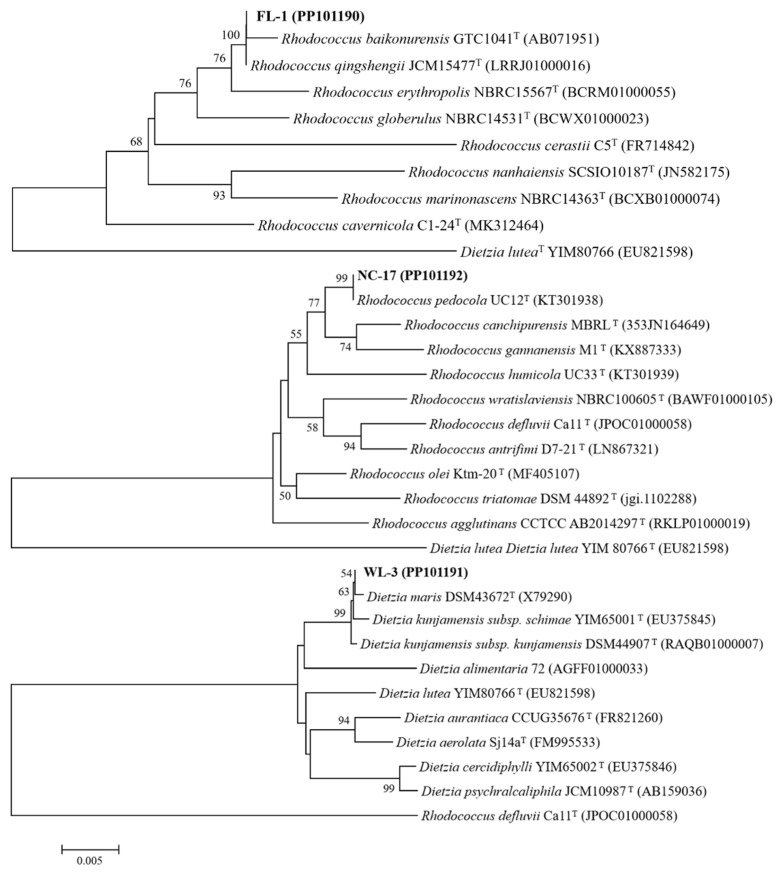
Phylogenetic tree of the three strains (FL-1, NC-17 and WL-3) based on 16S rDNA sequences. The calculations were performed according to a neighbour-joining algorithm (bootstrap number = 1000), and the scale bar represents 0.005 sequence divergence. The GenBank accession numbers are shown in parenthesis.

Three strains of high-efficiency petroleum hydrocarbon-degrading bacteria (FL-1, WL-3, and NC-17) were obtained through gradient dilution, enrichment cultivation, isolation, and purification. It could be found that there were significant differences and shared properties among the strains (Table 4). For instance, all three strains tested positive for Gram staining and glucose acid production while testing negative for gelatin liquefaction and indole production. Subsequently, the taxonomic identities of these three strains were determined by PCR amplification and comparison of the obtained 16S rDNA sequences with those available in the database. As shown in Figure 1, strains FL-1, NC-17, and WL-3 were homologous with *Rhodococcus baikonurensis* GTC 1041^T^, *Rhodococcus pedocola* UC12^T^, and *Dietzia maris* DSM 43672^T^, respectively, with a similarity of 100%, 99%, and 99%. All sequences of three bacteria were submitted to the Genetic Sequence Database at the National Center for Biotechnical Information (NCBI). The gene bank accession numbers of these strains in NCBI are PP101190 (for FL-1), PP101192 (for NC-17), and PP101191 (for WL-3). Based on the combined physiological and biochemical characteristics of the 16S rDNA analysis, strains FL-1 and NC-17 were finally identified as *Rhodococcus* sp., and strain WL-3 was identified as *Dietzia* sp.

### 3.3. Construction of a Bacterial Consortium

**Figure 2:**
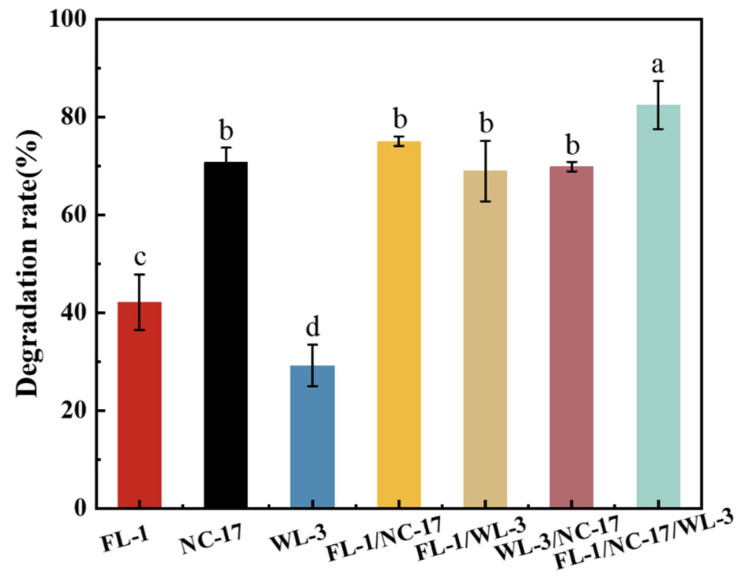
Degradation rate of petroleum hydrocarbons by bacterial strains and bacterial consortium. Different letters (a–d) above the bars indicate treatments with significant differences (*p* < 0.05).

Strains FL-1, NC-17, and WL-3 were selected to construct a bacterial consortium. Single and mixed bacteria were inoculated into diesel at a total inoculation rate of 2%. In the single-bacteria degradation experiment, strain NC-17 demonstrated the highest degradation rate, reaching 70.74% (Figure 2). Conversely, strain WL-3 exhibited the lowest degradation rate, with only 29.24% efficiency. In the mixed bacteria degradation experiment, the bacterial consortium composed of the three strains achieved the highest degradation rate of petroleum hydrocarbons, reaching 82.51%. The observed degradation rate of the bacterial consortium, composed of three strains, exceeded that of the consortium consisting of two strains. Moreover, it was 11.77% higher than the optimal degradation rate achieved by the single best-performing bacterium. The results showed that each strain within the bacterial consortium contributed to a synergistic degradation mechanism during the degradation process, thereby demonstrating superior degradation performance.

**Figure 3:**
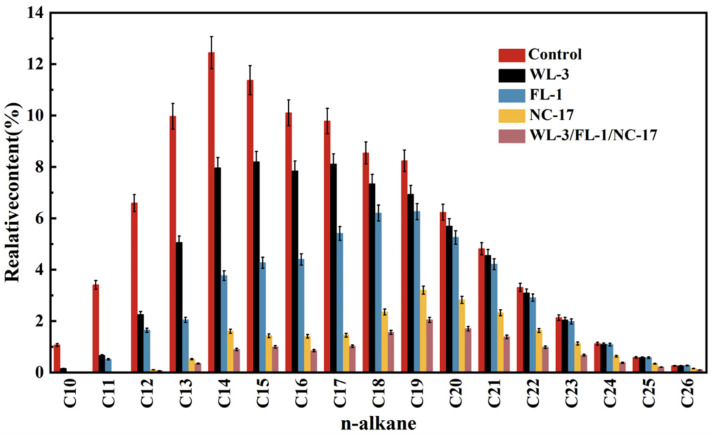
Variation of composition of n-alkanes in petroleum hydrocarbons.

To further investigate the synergistic degradation mechanism among strains, the residual n-alkanes after the degradation of petroleum hydrocarbons by single bacteria and the bacterial consortium were analyzed. As shown in Figure 3, the initial diesel’s n-alkane compositions were mainly C10–C20, with a relative content exceeding 5%. Previous studies have suggested that the carbon atom number range of short-chain alkanes, long-chain alkanes, and heavy long-chain alkanes is C10–C17, C18–C30, and C31–C38, respectively [35]. As shown in Figure 3, strains FL-1 and WL-3 exhibited degradation rates of 65.97% and 37.87% for short-chain alkanes (C10–C17), but only 18.4% and 10.36% for long-chain alkanes (C18–C26). The results indicated that strains FL-1 and WL-3 have a certain level of degradation capability for both long-chain and short-chain alkanes, albeit with a relatively weaker ability toward long-chain alkanes. In contrast, strain NC-17 exhibited an impressive degradation rate of 89.93% for short-chain alkanes and a significant 58.59% degradation for long-chain alkanes (Figure 3). The results indicated that strain NC-17 exhibits broad carbon chain utilization, establishing it as a highly efficient petroleum hydrocarbon-degrading strain. Previous research has noted that the degradation of simple alkanes in petroleum hydrocarbons occurs before the degradation of complex alkanes [36]. Therefore, the short-chain components had a higher degradation rate in this study. Additionally, different hydrocarbon structures and chain lengths require different degradation enzymes [37]. Previous studies reported that the ability of *Rhodococcus* sp. and *Dietzia* sp. to degrade hydrocarbons of various chain lengths is due to the presence of specific systems, such as CYP153 and alkB, responsible for encoding hydroxylase/oxygenase and other downstream enzymes [38,39]. Nie et al. confirmed a synergistic mechanism between CYP153 and alkB-like hydroxylases (alkW1) in the degradation of n-alkane by *Dietzia* sp. DQ12-45-1b. Specifically, CYP153 was responsible for hydroxylating n-alkanes shorter than C10, while alkW1 handled those longer than C14 [40]. Similarly, Binazadeh et al. found that *Rhodococcus* sp. Moj-3449 was preferred for degrading C14–C19 alkanes and C35 alkanes [41]. Seldin found that *Dietzia cinnamea* P4 was highly efficient in degrading the C11–C36 alkanes in crude oil, degrading about 88.11% of the C11–C23 alkanes after 10 d of incubation [42].

As shown in Figure 3, the bacterial consortium’s (NC-17/WL-3/FL-1) degradation rates for short-chain alkanes and long-chain alkanes were 93.57% and 74.38%, respectively, surpassing the degradation rates of individual bacteria. The result suggested a synergistic effect among the strains. Importantly, strain NC-17 solved the problem of the deficiency of strain FL-1 and strain WL-3 in the degradation ability of long-chain alkanes. Similarly, Tian et al. isolated a bacterial consortium composed of six *Bacillus* and *Pseudomonas* strains from heavy oil-polluted soil in the Bohai Bay area that had good petroleum hydrocarbon degradation efficiency (80.64%) [14]. Chen et al. combined two kinds of salt-tolerant and biosurfactant-producing bacteria, *Dietzia* sp. CN-3 and *Acinetobacter* sp. HC8-3S, to enhance degradation efficiency for various normal alkanes, cycloalkanes, branched alkanes, and aromatics compared to a single strain [43]. Due to the complex composition of petroleum hydrocarbons in shale gas OBDCs, the degradation of petroleum hydrocarbons by a single microorganism is limited [44]. Constructing a bacterial consortium for degradation harnesses the synergistic effects among strains, expanding the substrate spectrum for degradation, enhancing biodegradation efficiency, and improving adaptability to complex environments [45]. These findings underscore the promising potential of our bacterial consortium in environmental bioremediation, especially in addressing the challenges presented by shale gas drilling waste, which encompasses a diverse array of hydrocarbon components.

### 3.4. Optimization of Degradation Conditions

#### 3.4.1. Effect of Initial pH

**Figure 4:**
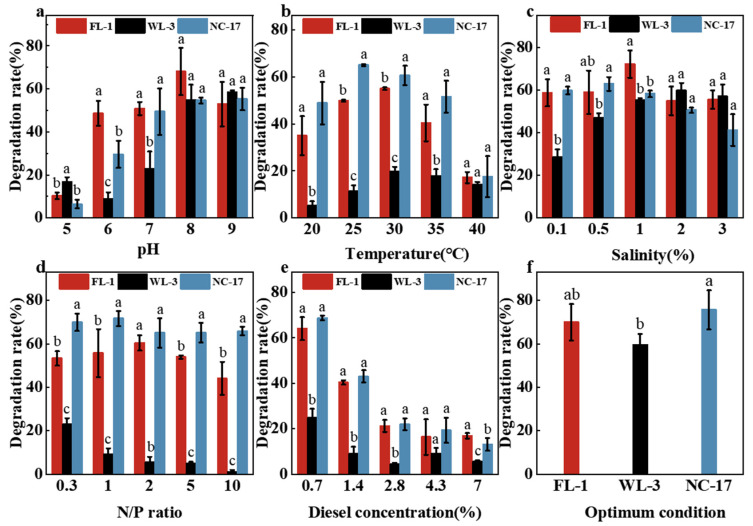
The degradation rate of diesel by these strains at different levels of (**a**) initial pH, (**b**) temperature, (**c**) salinity, (**d**) N/P ratio, (**e**) diesel concentration, and (**f**) optimum condition. Different letters (a–c) above the bars indicate treatments with significant differences (*p* < 0.05).

The pH directly influences bacteria’s growth, metabolism, and enzyme activities, making it a crucial factor in petroleum hydrocarbon degradation [46]. Figure 4a shows the effects of different initial pHs on the degradation efficiency of strains. According to Figure 4a, all three strains exhibited certain petroleum hydrocarbon degradation capabilities in the pH range of 5 to 9. The optimal pH for strain FL-1 was 8, while strains WL-3 and NC-17 demonstrated optimal performance at pH 9. While numerous studies have indicated that pH 7 is the optimal condition for petroleum hydrocarbons to degrade bacteria [18,47], the strains screened in this study exhibited the highest efficiency in degrading petroleum hydrocarbons within a weakly alkaline environment, indicating these strains have good alkaline resistance. Additionally, as the OBDCs samples were weakly alkaline (Table 1), it implies that these strains are well-suited for degrading petroleum hydrocarbons in OBDCs. Kavyanifard and Chen’s results also suggested the optimal crude oil degradation condition of *Dietzia* sp. and *Rhodococcus* sp. was pH 9, which was consistent with this study [32,48].

#### 3.4.2. Effect of Temperature

Temperature influences strain growth rates, enzyme activity, and the physical-chemical characteristics of petroleum hydrocarbons [49,50]. Therefore, temperature is considered an important factor in bioremediation. The influence of different temperatures on the degradation efficiency of the strains is shown in Figure 4b. With rising temperatures, the degradation efficiency of petroleum hydrocarbons by the three strains FL-1, NC-17, and WL-3 initially rises before subsequently declining. The preferred cultivation temperature for strains FL-1 and NC-17 was 30 °C, whereas strain WL-3 exhibited optimal growth at 25 °C. However, at 40 °C, the degradation rate of petroleum hydrocarbons dropped to only 17.60–20.99%, signifying a limited tolerance of these strains to higher temperatures. Lower temperatures can impede the initiation of biodegradation due to the high toxicity of low-molecular-weight toxic hydrocarbon pollutants with low volatility to microorganisms [51]. As the temperature rises, the solubility of short-chain hydrocarbons decreases, and the solubility of long-chain hydrocarbons increases. Concurrently, cell growth and metabolism accelerate, leading to enhanced surfactant production and the promotion of long-chain hydrocarbon degradation [18,46]. However, beyond a certain temperature threshold, the activity of degradation enzymes diminishes, and essential cellular components such as proteins and nucleic acids may incur irreversible damage [52].

#### 3.4.3. Effect of Salinity

Salinity directly affects the osmotic pressure of bacterial cells [53] and the metabolic level of degrading genes [54]. The effect of different salinities on the degradation efficiency of the strains is shown in Figure 4c. The results showed that different strains have different tolerances to salinity, and the optimal salinity for strains FL-1, WL-3, and NC-17 was 1%, 2%, and 0.5%, respectively. Notably, strains WL-3, FL-1, and NC-17 exhibited substantial petroleum hydrocarbon degradation rates (41.28–57.12%) even at a salinity of 3%, indicating their proficiency as petroleum hydrocarbon decomposers under elevated salinity conditions. Borzenkov et al. isolated two strains (*Rhodococcus* sp. and *Dietzia* sp.) from an oilfield, showcasing their ability to grow and produce biosurfactants under conditions of 0–10% salinity and a temperature range of 30–45 °C, using diesel as the sole carbon source [55]. This demonstrates the capability of certain microorganisms to thrive and degrade petroleum hydrocarbons even in environments with higher salinity.

#### 3.4.4. Effect of N/P Ratio

Nitrogen and phosphorus sources are essential elements for microbial growth and play a crucial role in the microbial degradation of hydrocarbon pollutants [37]. Figure 4d shows the effect of different N/P ratios on the degradation efficiency of petroleum hydrocarbons in the strains. As depicted, within the N/P ratio range of 0.3–10, strains FL-1 and NC-17 exhibited a trend of increasing and then decreasing petroleum hydrocarbon degradation rates, while strain WL-3 displayed a gradual decrease in degradation efficiency. The optimal N/P ratios of FL-1, NC-17, and WL-3 were 2, 1, and 0.3, respectively, which showed that the addition of nitrogen and phosphorus is not better and that there is an optimal match between microbial activity and the N/P ratio. Ouriache et al. found that when the C/N/P ratio was 60:2:1, the biodegradation efficiency of petroleum hydrocarbons was the highest. Conversely, an increase in the N/P ratio to 10 led to a significant decrease in biodegradation rates [56]. Previous studies have reported that changes in nitrogen source, C/N ratio, and organic nitrogen source can result in shifts in the biological activity of strains from one type of hydrocarbon to another [57]. It can be inferred from this study that an increase in the N/P ratio within a specific range may enhance the degradation of long-chain hydrocarbons.

#### 3.4.5. Effect of Diesel Concentration

Diesel was used as the sole carbon source to investigate the influence of different diesel concentrations on the degradation effect of petroleum hydrocarbons (Figure 4e). As depicted in Figure 4e, within the diesel concentration range of 0.7–7.0%, the degradation rate of petroleum hydrocarbons by the three strains decreased as the diesel concentration increased, with the optimal diesel addition being 0.7%. Exceeding this concentration led to an increase in the thickness of the oil film on the medium’s surface layer, thereby impeding material exchange between the degrading bacteria and the surrounding environment and intensifying stress on the degrading bacteria. Moreover, as the diesel concentration increases, an imbalance in the N/P ratio occurs [23], failing to meet the requirements for microbial degradation of petroleum hydrocarbons.

According to the above factor experiments, the optimum degradation conditions of the three strains were obtained (Table 5). To investigate the petroleum hydrocarbon degradation efficiency of the strains under their respective optimum conditions, diesel degradation experiments were carried out for 7 d, and the results are shown in Figure 4f. The degradation rates of petroleum hydrocarbons in FL-1, NC-17, and WL-3 were 69.87%, 75.61%, and 59.54%, respectively, surpassing those observed under non-optimal conditions. This indicates the significance of environmental factors in influencing the degradation of petroleum hydrocarbons. The optimal degradation conditions vary among different strains, showcasing that strains can exhibit enhanced petroleum hydrocarbon degradation capabilities under their respective optimal conditions.

### 3.5. Validation of the Degradation Performance of the Bacterial Consortium

**Figure 5:**
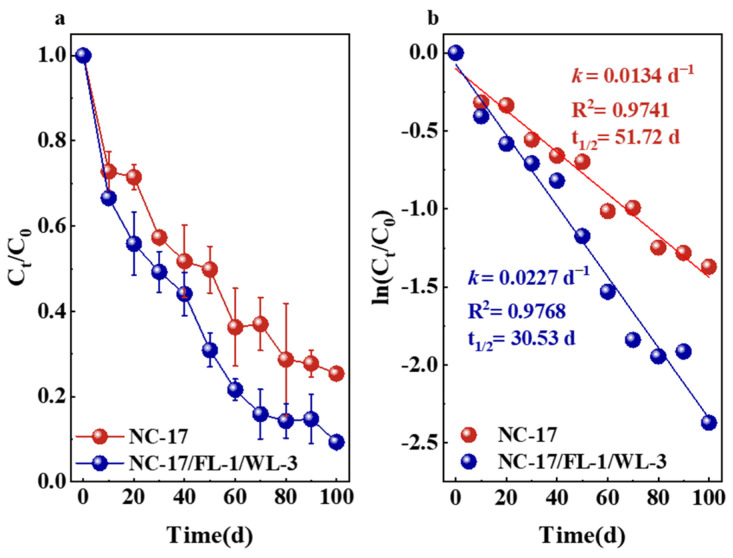
(**a**) The degradation rate of petroleum hydrocarbons with bioremediation time; (**b**) First-order kinetic fits of biodegradation process for petroleum hydrocarbons.

To validate the efficacy of the bacterial consortium in degrading petroleum hydrocarbons in practical OBDCs with 0.5% oil content, strain NC-17 and the bacterial consortium were employed for bioremediation. The degradation conditions of the bacteria consortium were set at pH 8, salinity 1%, temperature 30 °C, and N/P ratio 0.3. As shown in Figure 5a, after 100 d of bioremediation, the degradation rates of strain NC-17 and the bacterial consortium for petroleum hydrocarbons were 74.61% and 90.60%, respectively. To further assess the petroleum hydrocarbon degradation rate constants and half-life during degradation, the degradation kinetics data were fitted to a first-order kinetic equation (Figure 5b).

(2) $$\ln(C_{t}/C_{0}\;)=kt$$

(3) $$t_{1/2}=\ln(2\;)/k$$

where *C*_0_ and *C_t_* correspond to the initial and final levels of petroleum hydrocarbons before and after the validation experiment, respectively; *k* indicates the degradation rate constant; *t* indicates the experimental time; and *t*_1/2_ is the half-life of the petroleum hydrocarbons.

According to the findings of Rossetti et al., a higher rate constant signifies a more rapid biodegradation process, leading to a shorter half-life [58]. Analyzing the relationship between ln (*C_t_*/*C*_0_) and reaction time, it was determined that strain NC-17 exhibited a *k*-value of 0.0134 d^−1^ and a half-life of 51.72 d. Conversely, the bacterial consortium demonstrated a higher *k*-value (0.0227 d^−1^) and a shorter half-life (30.53 d). These outcomes highlight the markedly superior degradation efficiency of the bacterial consortium compared to individual bacteria, showcasing its effectiveness in degrading petroleum hydrocarbons in real OBDCs.

## 4. Conclusions

This study characterized the physical and chemical properties of OBDCs from shale gas Wells in FL, NC, WL, and DZ. The findings revealed contamination of the samples by hydrocarbons and heavy metals. Subsequently, three highly efficient petroleum hydrocarbon-degrading bacteria were isolated from the OBDC samples and identified as *Rhodococcus* sp. and *Dietzia* sp. GC analysis results demonstrated that the bacterial consortium, comprising the three strains, exhibited the highest petroleum hydrocarbon degradation rate. It achieved a degradation rate of 74.38% for long-chain hydrocarbons and 93.57% for short-chain hydrocarbons. At the same time, the optimum biodegradation temperature, pH, N/P ratio, diesel concentration, and salinity of each strain were determined through the experiment of optimizing degradation conditions. Finally, the degradation performance of petroleum hydrocarbons in actual OBDCs was verified. The degradation rate of petroleum hydrocarbons reached 90.60% after 100 d of biodegradation, and the degradation kinetic process was consistent with the first-order kinetic model. The method of multi-bacteria combined degradation of petroleum hydrocarbons proposed in this study provides a certain technical reserve for shale gas OBDC bioremediation technology.

## Figures and Tables

**Table 1:** Basic physical and chemical properties of OBDCs (%).

Sample	Oil Content	Water Content	Solid Content	pH
FL	11.16	9.80	75.95	9.75
WL	6.60	5.03	85.57	9.23
NC	10.50	14.05	73.60	9.24
DZ	8.43	9.57	78.33	9.62

**Table 2:** BTEX, PAHs, and TPHs in OBDCs (mg/kg).

Parameters	FL	WL	NC	DZ
Benzene	0.07	-	0.002	-
Toluene	0.73	-	0.09	0.03
Ethyl Benzene	1.05	-	0.59	0.06
Xylenes	6.33	-	4.71	0.49
PAHs	1.25	-	-	0.24
TPHs	2.67 × 10^4^	7.93 × 10^4^	5.47 × 10^4^	2.99 × 10^4^

**Table 3:** Concentration of metals in OBDCs (mg/kg).

Metals	FL	WL	NC	DZ
Barium (Ba)	2.21 × 10^5^	1.91 × 10^5^	1.91 × 10^5^	1.73 × 10^5^
Strontium (Sr)	2.29 × 10^3^	1.86 × 10^3^	1.97 × 10^3^	2.10 × 10^3^
Cadmium (Cd)	119.47	107.00	93.85	5.21
Arsenic (As)	12.30	16.8	7.43	11.95
Plumbum (Pb)	51.55	12.5	52.55	29.35
Chromium (Cr)	144.52	117.33	93.49	79.4
Cuprum (Cu)	71.62	67.16	76.55	83.8
Zinc (Zn)	267.75	140.30	232.00	400.50
Nickel (Ni)	45.90	37.13	49.00	49.95
Vanadium (V)	575.20	517.70	607.50	652.00
Manganese (Mn)	646.50	528.33	506.20	248.00
Cobalt (Co)	11.80	11.70	7.87	6.71
Titanium (Ti)	11.02	14.76	12.05	10.99

**Table 4:** Typical physiological and biochemical characteristics of the three strains.

Characteristics	FL-1	WL-3	NC-17
Colony form	Inerratic	Inerratic	Irregular
Cell shape	Bacilliform	Rounded	Bacilliform
Colony color	White	Yellow	White
Smoothness	Smooth	Smooth	Rough
Gram stain	+	+	+
Gelatin liquefaction	-	-	-
Hydrolysis of starch	-	-	-
Nitrate to nitrite	-	+	+
Indol test	-	-	-
Citrate test	+	-	+
Glucose fermentation	+	+	+

Symbols: + = growth positive, - = growth negative.

**Table 5:** The optimum degradation conditions of the three strains.

Strain	pH	Temperature (°C)	Salinity (%)	N/P Ratio	Diesel Concentration (%)
FL-1	8	30	1	2	0.7
WL-3	9	25	2	0.3	0.7
NC-17	9	30	0.5	1	0.7

## Data Availability

All genomic data were submitted to the GenBank NCBI database under accession numbers PP101190, PP101191 and PP101192. Data are contained within the article.

## References

[1] EPA Shale Gas Production Drives World Natural Gas Production Growth. https://www.eia.gov/todayinenergy/detail.php?id=275122016-815.

[2] Xie B., Qin J., Sun H., Wang S., Li X. (2022). Release characteristics of polycyclic aromatic hydrocarbons (PAHs) leaching from oil-based drilling cuttings. Chemosphere.

[3] Yang H., Diao H., Zhang Y., Xia S. (2022). Treatment and novel resource-utilization methods for shale gas oil based drill cuttings—A review. J. Environ. Manag..

[4] Ayati B., Molineux C., Newport D., Cheeseman C. (2019). Manufacture and performance of lightweight aggregate from waste drill cuttings. J. Clean. Prod..

[5] Liu P., Xiao Q., Dai N., Liu Z., Wang C. (2023). Study on Pyrolysis of Shale Gas Oil-Based Drilling Cuttings: Kinetics, Process Parameters, and Product Yield. ACS Omega.

[6] Hu Y., Mu S., Zhang J., Li Q. (2022). Regional distribution, properties, treatment technologies, and resource utilization of oil-based drilling cuttings: A review. Chemosphere.

[7] Kazamias G., Zorpas A.A. (2021). Drill cuttings waste management from oil & gas exploitation industries through end-of-waste criteria in the framework of circular economy strategy. J. Clean. Prod..

[8] Wu Y., Ding L., Zhang C., Shao T., Chen W. (2022). Experimental study on the treatment of oil-based drill cutting by pulsed dielectric barrier discharge plasma at atmospheric pressure. J. Clean. Prod..

[9] Khanpour R., Sheikhi-Kouhsar M.R., Esmaeilzadeh F., Mowla D. (2014). Removal of contaminants from polluted drilling mud using supercritical carbon dioxide extraction. J. Supercrit. Fluids.

[10] Yan P., Lu M., Guan Y., Zhang W., Zhang Z. (2011). Remediation of oil-based drill cuttings through a biosurfactant-based washing followed by a biodegradation treatment. Bioresour. Technol..

[11] Ball A., Stewart R., Schliephake K. (2011). A review of the current options for the treatment and safe disposal of drill cuttings. Waste Manag. Res. J. Int. Solid Wastes Public Clean. Assoc. ISWA.

[12] Hamidi Y., Ataei S.A., Sarrafi A. (2021). A highly efficient method with low energy and water consumption in biodegradation of total petroleum hydrocarbons of oily sludge. J. Environ. Manag..

[13] Bala S., Garg D., Thirumalesh B.V., Sharma M., Sridhar K., Inbaraj B.S., Tripathi M. (2022). Recent Strategies for Bioremediation of Emerging Pollutants: A Review for a Green and Sustainable Environment. Toxics.

[14] Tian X., Wang X., Peng S., Wang Z., Zhou R., Tian H. (2019). Isolation, screening, and crude oil degradation characteristics of hydrocarbons-degrading bacteria for treatment of oily wastewater. Water Sci. Technol..

[15] Ravi A., Ravuri M., Krishnan R., Narenkumar J., Anu K., Alsalhi M.S., Devanesan S., Kamala-Kannan S., Rajasekar A. (2022). Characterization of petroleum degrading bacteria and its optimization conditions on effective utilization of petroleum hydrocarbons. Microbiol. Res..

[16] Viesser J.A., Sugai-Guerios M.H., Malucelli L.C., Pincerati M.R., Karp S.G., Maranho L.T. (2020). Petroleum-Tolerant Rhizospheric Bacteria: Isolation, Characterization and Bioremediation Potential. Sci. Rep..

[17] Nicolau E., Kuhn L., Marchal R., Jouanneau Y. (2009). Proteomic investigation of enzymes involved in 2-ethylhexyl nitrate biodegradation in Mycobacterium austroafricanum IFP 2173. Res. Microbiol..

[18] Liu B., Ju M., Liu J., Wu W., Li X. (2016). Isolation, identification, and crude oil degradation characteristics of a high-temperature, hydrocarbon-degrading strain. Mar. Pollut. Bull..

[19] Xia W., Li J., Xia Y., Song Z., Zhou J. (2012). Optimization of diesel oil biodegradation in seawater using statistical experimental methodology. Water Sci. Technol. A J. Int. Assoc. Water Pollut. Res..

[20] Nayak A.S., Vijaykumar M.H., Karegoudar T.B. (2009). Characterization of biosurfactant produced by *Pseudoxanthomonas* sp. PNK-04 and its application in bioremediation. Int. Biodeterior. Biodegrad..

[21] Zhang K., Hua X.-F., Han H.-L., Wang J., Miao C.-C., Xu Y.-Y., Huang Z.-D., Zhang H., Yang J.-M., Jin W.-B. (2008). Enhanced bioaugmentation of petroleum- and salt-contaminated soil using wheat straw. Chemosphere.

[22] Khan M.A.I., Biswas B., Smith E., Naidu R., Megharaj M. (2018). Toxicity assessment of fresh and weathered petroleum hydrocarbons in contaminated soil—A review. Chemosphere.

[23] Ghorbannezhad H., Moghimi H., Dastgheib S.M.M. (2022). Biodegradation of high molecular weight hydrocarbons under saline condition by halotolerant Bacillus subtilis and its mixed cultures with Pseudomonas species. Sci. Rep..

[24] Liu Y., Li C., Huang L., He Y., Zhao T., Han B., Jia X. (2017). Combination of a crude oil-degrading bacterial consortium under the guidance of strain tolerance and a pilot-scale degradation test. Chin. J. Chem. Eng..

[25] Rasti A., Ameri A., Riahi M.A. (2021). Aerobic degradation of oil-based mud drilling fluid by in situ bacteria in the Hawizeh Marshes. J. Pet. Explor. Prod. Technol..

[26] Zhang B., Shan B., Zhao Y., Zhang L. (2020). Review of Formation and Gas Characteristics in Shale Gas Reservoirs. Energies.

[27] Kogbara R.B., Ayotamuno J.M., Onuomah I., Ehio V., Damka T.D. (2016). Stabilisation/solidification and bioaugmentation treatment of petroleum drill cuttings. Appl. Geochem..

[28] Holt S.G., Kriey N.R., Sneath P.H.A. (1998). Bergy’s Manual of Determinative for Bacteriology.

[29] Ding Z., Wu J., You A., Huang B., Cao C. (2017). Effects of heavy metals on soil microbial community structure and diversity in the rice (*Oryza sativa* L. subsp. Japonica, Food Crops Institute of Jiangsu Academy of Agricultural Sciences) rhizosphere. Soil Sci. Plant Nutr..

[30] Li C., Quan Q., Gan Y., Dong J., Fang J., Wang L., Liu J. (2020). Effects of heavy metals on microbial communities in sediments and establishment of bioindicators based on microbial taxa and function for environmental monitoring and management. Sci. Total Environ..

[31] Al-Marri S., Eldos H.I., Ashfaq M.Y., Saeed S., Skariah S., Varghese L., Mohamoud Y.A., Sultan A.A., Raja M.M. (2023). Isolation, identification, and screening of biosurfactant-producing and hydrocarbon-degrading bacteria from oil and gas industrial waste. Biotechnol. Rep..

[32] Kavyanifard A., Ebrahimipour G., Ghasempour A. (2015). Optimization of crude oil degradation by *Dietzia cinnamea* KA1, capable of biosurfactant production. J. Basic Microbiol..

[33] Makkar R.S., Cameotra S.S. (2002). Effects of various nutritional supplements on biosurfactant production by a strain of Bacillus subtilis at 45 °C. J. Surfactants Deterg..

[34] Kiran G.S., Nishanth L.A., Priyadharshini S., Anitha K., Selvin J. (2014). Effect of Fe nanoparticle on growth and glycolipid biosurfactant production under solid state culture by marine Nocardiopsissp. MSA13A. BMC Biotechnol..

[35] Li C., Zhou Z.-X., Jia X.-Q., Chen Y., Liu J., Wen J.-P. (2013). Biodegradation of Crude Oil by a Newly Isolated Strain *Rhodococcus* sp. JZX-01. Appl. Biochem. Biotechnol..

[36] Nkem B.M., Halimoon N., Yusoff F.M., Johari W.L.W., Zakaria M.P., Medipally S.R., Kannan N. (2016). Isolation, identification and diesel-oil biodegradation capacities of indigenous hydrocarbon-degrading strains of Cellulosimicrobium cellulans and Acinetobacter baumannii from tarball at Terengganu beach, Malaysia. Mar. Pollut. Bull..

[37] Das N., Chandran P. (2011). Microbial Degradation of Petroleum Hydrocarbon Contaminants: An Overview. Biotechnol. Res. Int..

[38] Roslee A.F.A., Zakaria N.N., Convey P., Zulkharnain A., Lee G.L.Y., Gomez-Fuentes C., Ahmad S.A. (2020). Statistical optimisation of growth conditions and diesel degradation by the Antarctic bacterium, *Rhodococcus* sp. strain AQ5–07. Extremophiles.

[39] Liang J.-L., JiangYang J.-H., Nie Y., Wu X.-L. (2016). Regulation of the Alkane Hydroxylase CYP153 Gene in a Gram-Positive Alkane-Degrading Bacterium, Dietzia sp. Strain DQ12-45-1b. Appl. Environ. Microbiol..

[40] Nie Y., Liang J.-L., Fang H., Tang Y.-Q., Wu X.-L. (2014). Characterization of a CYP153 alkane hydroxylase gene in a Gram-positive Dietzia sp. DQ12-45-1b and its “team role” with alkW1 in alkane degradation. Appl. Microbiol. Biotechnol..

[41] Binazadeh M., Karimi I.A., Li Z. (2009). Fast biodegradation of long chain n-alkanes and crude oil at high concentrations with *Rhodococcus* sp. Moj-3449. Enzym. Microb. Technol..

[42] von der Weid I., Marques J.M., Cunha C.D., Lippi R.K., dos Santos S.C.C., Rosado A.S., Lins U., Seldin L. (2007). Identification and biodegradation potential of a novel strain of *Dietzia cinnamea* isolated from a petroleum-contaminated tropical soil. Syst. Appl. Microbiol..

[43] Chen W., Kong Y., Li J., Sun Y., Min J., Hu X. (2020). Enhanced biodegradation of crude oil by constructed bacterial consortium comprising salt-tolerant petroleum degraders and biosurfactant producers. Int. Biodeterior. Biodegrad..

[44] Li X., Zhao L., Adam M. (2016). Biodegradation of marine crude oil pollution using a salt-tolerant bacterial consortium isolated from Bohai Bay, China. Mar. Pollut. Bull..

[45] Phulpoto I.A., Hu B., Wang Y., Ndayisenga F., Li J., Yu Z. (2021). Effect of natural microbiome and culturable biosurfactants-producing bacterial consortia of freshwater lake on petroleum-hydrocarbon degradation. Sci. Total Environ..

[46] Singh K., Chandra S. (2014). Treatment of Petroleum Hydrocarbon Polluted Environment Through Bioremediation: A Review. Pak. J. Biol. Sci. PJBS.

[47] Van Hong Thi P., Chaudhary D.K., Jeong S.-W., Kim J. (2018). Oil-degrading properties of a psychrotolerant bacterial strain, *Rhodococcus* sp. Y2-2, in liquid and soil media. World J. Microbiol. Biotechnol..

[48] Chen X., Shan G., Shen J., Zhang F., Liu Y., Cui C. (2023). In situ bioremediation of petroleum hydrocarbon–contaminated soil: Isolation and application of a Rhodococcus strain. Int. Microbiol..

[49] Koshlaf E., Ball A. (2017). Soil bioremediation approaches for petroleum hydrocarbon polluted environments. AIMS Microbiol..

[50] Adams G., Tawari-Fufeyin P., Okoro S., Ehinomen I. (2015). Bioremediation, Biostimulation and Bioaugmention: A Review. Int. J. Environ. Bioremediat. Biodegrad..

[51] Dindar E., Topaç F.O., BaŞKaya H. (2016). Biodegradation of used engine oil in a wastewater sludge-amended agricultural soil. Turk. J. Agric. For..

[52] Ferreira T., Coelho M.A., Rocha-Leão M.H. (2012). Factors influencing crude oil biodegradation by Yarrowia lipolytica. Braz. Arch. Biol. Technol..

[53] Gatti M., García-Usach F., Seco A., Ferrer J. (2010). Wastewater COD Characterization: Analysis of Respirometric and Physical-Chemical Methods for Determining Biodegradable Organic Matter Fractions. J. Chem. Technol. Biotechnol..

[54] Wang C., Huang Y., Zhang Z., Wang H. (2018). Salinity effect on the metabolic pathway and microbial function in phenanthrene degradation by a halophilic consortium. AMB Express.

[55] Borzenkov I.A., Milekhina E., Gotoeva M., Rozanova E., Beliaev S. (2006). The properties of hydrocarbon-oxidizing bacteria isolated from the oilfields of Tatarstan, Western Siberia, and Vietnam. Mikrobiologiia.

[56] Ouriache H., Moumed I., Arrar J., Abdelkader N., Lounici H. (2020). Influence of C/N/P ratio evolution on biodegradation of petroleum hydrocarbons-contaminated soil. Alger. J. Environ. Sci. Technol..

[57] Al Disi Z., Jaoua S., Al-Thani D., Almeer S., Zouari N. Isolation, Screening and Activity of Hydrocarbon-Degrading Bacteria from Harsh Soils. Proceedings of the World Congress on Civil, Structural, and Environmental Engineering.

[58] Rossetti I., Conte F., Ramis G. (2021). Kinetic Modelling of Biodegradability Data of Commercial Polymers Obtained under Aerobic Composting Conditions. Eng.

